# Clinical outcomes of seasonal influenza and pandemic influenza A (H1N1) in pediatric inpatients

**DOI:** 10.1186/1471-2431-10-72

**Published:** 2010-10-06

**Authors:** Pranita D Tamma, Alison E Turnbull, Aaron M Milstone, Sara E Cosgrove, Alexandra Valsamakis, Alicia Budd, Trish M Perl

**Affiliations:** 1 Department of Pediatric Infectious Diseases, The Johns Hopkins Medical Institution, 200 North Wolfe Street, Suite 3150 Baltimore, Maryland, 21287, USA; 2 Department of Epidemiology, Johns Hopkins University Bloomberg School of Public Health, 615 N. Wolfe Street Baltimore, Maryland, 21287, USA; 3 Department of Infectious Diseases, The Johns Hopkins Medical Institution, 600 North Wolfe Street, Osler 424 Baltimore, Maryland, 21287, USA; 4 Department of Pathology, The Johns Hopkins Medical Institution, Meyer B1-193, 600 North Wolfe Street Baltimore, Maryland, 21287, USA; 5 Department of Infection Control, The Johns Hopkins Medical Institution, 600 North Wolfe Street, Osler 424 Baltimore, Maryland, 21287, USA; 6 Department of Infectious Diseases, The Johns Hopkins Medical Institution, 600 North Wolfe Street, Osler 424, Baltimore, Maryland, 21287, USA

## Abstract

**Background:**

In April 2009, a novel influenza A H1N1 (nH1N1) virus emerged and spread rapidly worldwide. News of the pandemic led to a heightened awareness of the consequences of influenza and generally resulted in enhanced infection control practices and strengthened vaccination efforts for both healthcare workers and the general population. Seasonal influenza (SI) illness in the pediatric population has been previously shown to result in significant morbidity, mortality, and substantial hospital resource utilization. Although influenza pandemics have the possibility of resulting in considerable illness, we must not ignore the impact that we can experience annually with SI.

**Methods:**

We compared the outcomes of pediatric patients ≤18 years of age at a large urban hospital with laboratory confirmed influenza and an influenza-like illness (ILI) during the 2009 pandemic and two prior influenza seasons. The primary outcome measure was hospital length of stay (LOS). All variables potentially associated with LOS based on univariable analysis, previous studies, or hypothesized relationships were included in the regression models to ensure adjustment for their effects.

**Results:**

There were 133 pediatric cases of nH1N1 admitted during 2009 and 133 cases of SI admitted during the prior 2 influenza seasons (2007-8 and 2008-9). Thirty-six percent of children with SI and 18% of children with nH1N1 had no preexisting medical conditions (p = 0.14). Children admitted with SI had 1.73 times longer adjusted LOS than children admitted for nH1N1 (95% CI 1.35 - 2.13). There was a trend towards more children with SI requiring mechanical ventilation compared with nH1N1 (16 vs.7, p = 0.08).

**Conclusions:**

This study strengthens the growing body of evidence demonstrating that SI results in significant morbidity in the pediatric population. Pandemic H1N1 received considerable attention with strong media messages urging people to undergo vaccination and encouraging improved infection control efforts. We believe that this attention should become an annual effort for SI. Strong unified messages from health care providers and the media encouraging influenza vaccination will likely prove very useful in averting some of the morbidity related to influenza for future epidemics.

## Background

In April 2009, a novel influenza A H1N1 (nH1N1) virus emerged and spread rapidly worldwide. News of the pandemic led to a heightened awareness of the consequences of influenza, as well as some apprehension, in both the general population, the public health sector, and among healthcare providers. Concerns regarding the potential impact of this novel influenza strain led to enhanced infection control practices and strengthened vaccination efforts for both healthcare workers and the general population." [1-5] The media was actively involved in informing the public of the potential consequences of influenza infection and encouraging vigorous vaccination efforts [6].

Importantly, seasonal influenza (SI) illness in the pediatric population has been previously shown to result in significant morbidity, mortality, as well as substantial hospital resource utilization [7-10]. Although influenza pandemics have the possibility of resulting in considerable illness, we cannot ignore the impact we experience annually with SI. Perhaps the aggressive campaigning and resource allocation reserved for pandemic influenza should be emphasized on an annual basis for SI. We compared the outcomes of a large cohort of pediatric patients admitted for nH1N1 infection to children admitted with SI in the 2007-8 and 2008-9 influenza seasons to determine if there was a significant difference in morbidity. We hypothesized that children admitted with SI would have outcomes including admission to the pediatric intensive care unit, presence of bacterial superinfections, and hospital length of stay (LOS) similar to those admitted with nH1N1.

## Methods

### Setting

This is a retrospective, cohort study conducted at The Johns Hopkins Children's Medical and Surgical Center (JHCMSC), a part of the Johns Hopkins Hospital (JHH). The JHCMSC is 175-bed pediatric teaching hospital located in Baltimore, Maryland that provides medical care to the Baltimore community and also serves as a tertiary care pediatric hospital for the surrounding region. The hospital admits >8500 children annually, of which >1600 are cared for in the 26-bed pediatric intensive care unit (PICU).

### Study Period and Population

We included all children and adolescents 18 years of age and younger admitted to the JHCMSC with a laboratory-confirmed diagnosis of influenza within 24 hours of admission and an influenza-like illness (ILI). An ILI was defined as a fever and upper respiratory tract symptoms (cough, sore throat, rhinorrhea, congestion), lower respiratory symptoms (wheezing, chest pain, shortness of breath), or gastrointestinal symptoms (abdominal pain, vomiting, diarrhea). There were no exclusion criteria. Influenza cases during the 2007-9 seasons that met the inclusion criteria were evaluated. The nH1N1 cases were compared to SI cases.

The Hospital Epidemiology and Infection Control (HEIC) Department has an active surveillance program for respiratory infections among all patients presenting to JHH. http://www.hopkinsmedicine.org/heic/ID/h1n1/index.html. All children with ILI admitted to the JHCMSC are sampled for respiratory viruses. Results are prospectively recorded into the electronic patient medical record and simultaneously supplied to the HEIC computer surveillance system (TheraDoc, Inc, Salt Lake City, UT). One Infection Control Practitioner (AB) queries the system prospectively to identify all patients with positive influenza testing and additional data are abstracted and included in a database. Pediatric cases were identified from the master HEIC database. Medical records were reviewed and demographic, laboratory, radiographic, and clinical data were collected onto standardized case report forms. Patients were identified as having bacterial pneumonia based on previously described criteria including radiographic evidence of a new infiltrate [11].

### Laboratory Methods

All respiratory virus samples were collected via nasopharyngeal aspirate (NPA) by trained nursing staff. Respiratory virus testing from May-December 2009 consisted of direct fluorescent antibody (DFA) assay (D^3^, Diagnostic Hybrids Inc.[DHI], Athens, OH). Shell vial (R-Mix Too, DHI) and conventional tube cultures (rhesus monkey kidney and A549 cells, Diagnostic Hybrids, Inc.) were inoculated in parallel with DFA for all samples. Rapid immuno-card tests (BINAX NOW Influenza A and B, Inverness Medical, Princeton, NJ) were performed initially on all NPA samples from December - April of 2007-8 and 2008-9. No further testing was performed on positive samples; DFA, shell vial, and tube culture were performed on all samples with a negative immuno-card result.

From May through December 2009, all influenza A positive samples from admitted children were sent to The Maryland Department of Health and Mental Hygiene and confirmed by real-time reverse transcriptase-PCR as novel influenza A (H1N1) as described by the Centers for Disease Control and Prevention (CDC) [12].

### Statistical Analysis

Statistical analyses were performed using Stata version 10.0 (STATA Corp., College Station, TX) and the R statistical package (version 2.10.1). Comparisons between patients with seasonal influenza and nH1N1 influenza were performed using Fisher's exact test for non-parametric data and Student's t-test with unequal variances for continuous variables. Hospital LOS was log transformed to achieve a normal distribution and analyzed using linear regression and Poisson regression models. Models including interaction terms and excluding high leverage observations were fitted and residual analysis including residuals plots, Q-Q plots, and added variable plots were examined for violations of regression model assumptions. All variables potentially associated with LOS based on univariable analysis, previous studies, or hypothesized relationships were included in the final linear model to ensure adjustment for their effects. Two-sided p-values of < 0.05were treated as statistically significant for all tests. The Johns Hopkins University Institutional Review Board approved the study with a waiver of informed consent.

## Results

**Figure 1:**
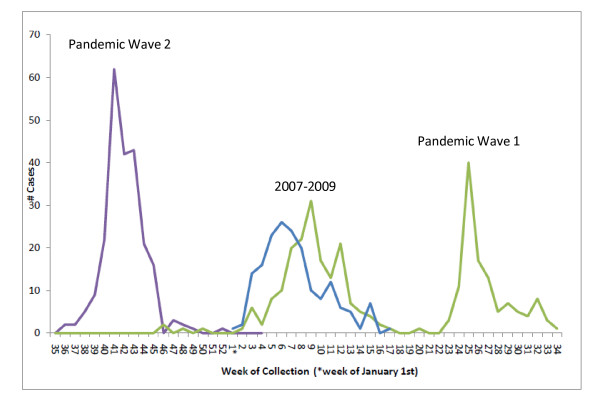
**Epidemic curve of pandemic influenza A H1N1 and seasonal influenza (2007-2008 and 2008-2009) for children admitted to The Johns Hopkins Children's Medical and Surgical Center**.

We identified 133 pediatric patients with a laboratory-confirmed diagnosis of seasonal influenza during the 2007-9 seasons. Coincidentally, we identified 133 children with laboratory-confirmed nH1N1 who met our inclusion criteria. The first confirmed nH1N1 virus infection at JHCMSC occurred on May 1^st^, 2009 and the last case included in this study was diagnosed on November 25^th^, 2009. Figure 1 depicts the epidemic curve of nH1N1 and seasonal influenza during the prior two epidemic periods at JHCMSC. Similar proportions of children with positive laboratory testing for influenza in the emergency room were admitted to JHCMSC during the nH1N1 pandemic and prior influenza seasons (38% vs. 47%, p = 0.12 ). There was no difference in the percentage of hospitalized patients admitted directly to the PICU (19% vs. 18%, p = .99).

We aggregated SI data from the two prior seasons because patients in the 2007-8 and 2008-9 seasons were not significantly different in terms of demographic features, underlying medical conditions, clinical course, and outcomes (data not shown). All patients had valid reasons for admission including hypoxia, respiratory distress, shock requiring pressors, immunocompromised and febrile, neonates with fever, or sickle cell disease with fever. These patient types were equally distributed between the seasons (p ≥ 0.17).

### Demographics and presenting symptoms

Children admitted with SI and nH1N1 influenza were similar with regards to age, race, and gender (Table 1). Presenting symptoms were also similar among patients with SI and nH1N1 influenza infection (Table 1); except seizures were reported in 19 (14%) of the children with SI and 5 (4%) of the children with nH1N1 (p = 0.004). Eleven of the children admitted for SI and 4 of the children admitted for nH1N1 who presented with seizures had no known underlying seizure disorder.

**Table 1:** Demographic characteristics, presenting symptoms, and diagnostic testing of children admitted with seasonal influenza (2007-2009) and 2009 novel influenza A (nH1N1) infection

	Seasonal influenza	nH1N1 influenza	p-value
**Demographic characteristics**	133	133	

**Age**			

Mean (SD)	7.0 (5.7)	7.3 (5.4)	.64

Median	5.0	6.0	

**Race**			.43

African American	76 (57%)	83 (62%)	

Asian	2 (2%)	5 (4%)	

Caucasian	47 (35%)	37 (28%)	

Hispanic	8 (6%)	8 (6%)	

**Gender**			.81

Male	67 (50%)	70 (53%)	

Female	66 (50%)	63 (47%)	

**Presenting Symptoms**^†^			

Upper respiratory tract	110 (83%)	116 (87%)	.39

Lower respiratory tract	55 (41%)	61 (46%)	.50

Gastrointestinal	35 (26%)	43 (32%)	.35

Mylagias	14 (11%)	8 (6%)	27

Headaches	11 (8%)	18 (14%)	.24

Seizures	19 (14%)	5 (4%)	< .01

Lethargy	33 (25%)	40 (30%)	.41

**Diagnostic Testing**^††^			

Rapid immuno-card^†††^	55 (41.4%)	N/A	

Direct fluorescent antibody	78(33%)	39 (29%)	.39

Shell vial culture	36 (46%)	90 (68%)	.02
Tube culture	16 (21%)	4(3%)	.40

^† ^Fever was part of inclusion criteria, ^††^By 1^st ^positive test, ^†††^If positive for seasonal influenza no further testing performed

### Preexisting Medical Conditions

Thirty-five (26%) children admitted with seasonal influenza and 24 (18%) patients with nH1N1 had no preexisting medical conditions (p = 0.14). Twenty (15%) SI and 40 (30%) nH1N1 admissions had an underlying diagnosis of asthma (p < 0.01). Neuromuscular disorders were more prevalent in children admitted with SI compared with nH1N1 (18% vs.7%, p < 0.01). There were otherwise no significant differences with regards to underlying medical conditions between the two groups. (Table 2)

**Table 2:** Preexisting medical conditions among children admitted with seasonal influenza (2007-2009) and novel influenza A H1N1 (nH1N1) infection

	Seasonal influenza	nH1N1 influenza	p-value
**None**	35 (26%)	24(18%)	.14

**Obesity**^†^	49 (46%)	51(45%)	1.00

**Asthma**	20 (15%)	40 (30%)	< .01

**Sickle Cell Disease**	17 (12.8%)	26 (20%)	.18

**Neuromuscular Disorder**	24 (18%)	9 (7%)	< .01

**Diabetes Mellitus (type 1)**	2 (2%)	2 (2%)	1.00

**Chronic Lung Disease**	15 (11%)	15 (11%)	1.00

**Malignancy**	13 (10%)	11(8%)	.83

**Immunodeficiency**^††^	10 (8%)	4 (3%)	.17

**Prematurity (24-37 w)**	5 (4%)	10 (8%)	.29

**Cardiac disorder**	8 (6%)	5 (4%)	.57

**Metabolic disorder**	7 (5%)	7 (5%)	1.00

**Renal Failure**	3 (2%)	1 (1%)	.62

**Cystic Fibrosis**	4 (3%)	3 (2%)	1.00

^†^Body mass index ≥90%, ^††^HIV, solid organ transplant, immunodeficiency, corticosteroids ≥ 2 mg/kg/day for >14 days

### Antimicrobial use

Based on CDC guidelines, and in contrast to SI epidemics, antiviral therapy was recommended for all admitted patients with nH1N1 [5]. Thirty-seven (28%) children with SI and 106 (80%) children admitted with nH1N1 received antiviral therapy with activity against influenza (p < 0.001). When stratifying LOS based on influenza type and antiviral therapy, LOS was not modified. (Table 3) Chest imaging was performed on a significantly higher proportion of children with nH1N1 compared with SI (92% vs. 83%, p = 0.03). However, similar percentages of children in both groups met diagnostic criteria for bacterial pneumonia (23% vs. 23%). Interestingly, 61 (72%) of nH1N1 and 37 (36%) SI children, received treatment courses of antibiotics for a diagnosis of "bacterial pneumonia" (p < 0.01).

**Table 3:** The timing of antiviral therapy and length of stay among children admitted with seasonal influenza (2007-2009) and novel H1N1 influenza (nH1N1)

	Seasonal influenza **LOS**^**†**^	nH1N1 influenza **LOS**^**†**^	p-value
**No antiviral therapy**	7.2 days (n = 92)	3.6 days (n = 27)	< .01

**Antiviral therapy**	6.7 days (n = 37)	4.4 days (n = 106)	.03

**Antiviral therapy ≥48 hours after symptom onset or no antiviral therapy**	7.2 days (n = 120)	4.5 days (n = 82)	< .01

**Antiviral therapy ≤48 hours of symptom onset**	5.6 days (n = 12)	3.7 days (n = 51)	.11

^† ^Median length of stay, (n = number in each sample)

Ten children admitted for SI had bacteremia. Blood cultures grew methicillin-resistant *Staphylococcus aureus *in 3 children, methicillin-susceptible *Staphylococcus aureus *in another 3 children, and *Streptococcus pneumoniae *in 3 children. One child grew *Streptococcus pyogenes *in a blood culture. No bacteremia was identified in any of the children with nH1N1.

### Outcomes

The median LOS for SI was 2 days longer than nH1N1; 5 days [IQR 3-7] vs. 3 days [IQR 2-4]. (Table 4) One patient in the SI cohort had a LOS of 254 days due to complications unrelated to influenza; however, mean LOS remained significant after this outlier was removed (p < 0.001). After adjusting for age, race, gender, number of co-morbid illnesses, presence of a neuromuscular disorder, asthma, obesity, presence of an infiltrate concerning for bacterial pneumonia, antiviral therapy, and >48 hours of antibiotic use, children with SI had 1.73 times longer LOS (95% CI 1.35 - 2.13) than children with nH1N1.

**Table 4:** Non-pharmacologic treatments and outcomes of children admitted with seasonal influenza (2007-2009) and novel influenza A H1N1 (nH1N1)

	Seasonal influenza	nH1N1 influenza	p-value
**Length of Stay**			0.02
Mean (SD)	8.9 (23.0) days	4.2 (4.8) days	
Median (Range)	5 (1 - 254)	3 (1 -32)	

**PICU admission**	37 (28%)	27 (20%)	0.20

**Need for noninvasive positive pressure ventilation**	16 (12%)	11 (8%)	0.31

**Need for mechanical ventilation**	16 (12%)	7 (5%)	0.08

**Days of oxygen**			0.14
Mean (SD)	8.7 (19.3)	4.2 (5.3)	
Median (Range)	4 (1 -140)	2.5 (1-30)	

**Days of positive pressure ventilation or mechanical ventilation**			0.74
Mean (SD)	7.04 (1.71)	6.14(1.87)	
Median (Range)	5(1-38)	2.5 (1-24)	

**PICU length of stay**			0.20
Mean (SD)	10.9 (29.3) days	4.5 (5.7) days	
Median (Range)	4 (1- 179)	2 (1 - 26)	

Thirty-seven (28%) SI cases and 27 (20%) nH1N1 cases (p = 0.20) were admitted to the PICU. Sixteen (12%) of the SI children required noninvasive positive pressure ventilation compared with 11(8%) of the nH1N1 children (p = 0.31). There was a trend towards more children with SI requiring mechanical ventilation compared with nH1N1 (16 vs.7, respectively p = 0.08). One patient, with no underlying medical conditions, diagnosed with SI required extracorporeal membrane oxygenation. This patient subsequently died. There were no deaths in children admitted with nH1N1.

## Discussion

It is critical to understand outcomes for infections to ensure appropriate planning and utilization of healthcare resources. We compared the clinical outcomes of nH1N1 to those of SI in a large cohort of hospitalized children. We found that children admitted with epidemic influenza over the past two influenza seasons (2007-9) appeared to have a longer adjusted LOS, with a median of almost 2 more days of hospitalization than children admitted for nH1N1. Median LOS in our population of children was somewhat higher than reported in other retrospective cohort studies and may be related to the higher percentage of children with high-risk medical conditions in our cohort compared with other studies[10,13].

Despite initial concerns about severity of disease expected for pandemic nH1N1, our data demonstrates that outcomes among patients with SI are at least as concerning. This is particularly relevant as there was a concerted effort to improve infection prevention and control practices, immunization use, and bed allotment for the anticipated large numbers of critically ill children with nH1N1[1-6]. Based on results from our study, some of these practices may need to be sustained on an annual basis for SI.

The significance of SI appears to be underappreciated by the general population and the health care community. Although the majority of epidemic influenza virus infection is usually mild, influenza has the potential to cause severe disease, particularly in young children and children with underlying medical conditions [14,15]. Children are disproportionately affected by hospitalization rates during SI epidemics. A review of a national database found that the relative risk (RR) for an influenza-associated hospitalization relative to death was estimated to be 270 for children 5 years of age and under, compared to 11 amongst adults aged 50-64 [8]. While mortality is often used to estimate the effect of influenza among adults, it may be an insensitive indicator of the effect of influenza in children as the primarily burden is related to its morbidity [16,17].

Among healthy-children, oseltamivir has been shown to reduce median duration of influenza illness by 36 hours [18]. Although the decreased LOS with nH1N1 could be attributed to wider antiviral use in this population, our data does not support this interpretation. After stratifying by antiviral therapy and influenza type, the difference in LOS between SI and nH1N1 pediatric admissions persisted and antiviral therapy did not appear to significantly influence LOS in our study. Prospective data are needed to define the relative benefits of early antiviral therapy in hospitalized pediatric patients.

In both SI and nH1N1 patients in our cohort, antibiotics were prescribed for bacterial pneumonia when criteria for this diagnosis were not fulfilled. Almost 50% of patients with nH1N1 who received antibiotics for a diagnosis of "bacterial pneumonia" did not meet clinical criteria. Unnecessary prescription of antibiotics may have been related to the general apprehension in the health care community with regards to pandemic nH1N1. Antibiotics have the potential for associated toxicities, costs, and the development of multi-drug resistant organisms. Future studies are needed to better determine the criteria for antibiotics for influenza-related bacterial complications.

During the nH1N1 pandemic, there were widespread media efforts to educate the public about potential consequences of influenza and need for vaccination, especially for the most high-risk groups. Given that annual influenza vaccination is the most effective method for preventing influenza virus infection and its complications, additional efforts need to be made to ensure annual SI vaccination of all children, particularly children at higher risk for influenza complications [8,9,14,15,19-22]. Our results emphasize the need for vaccination against seasonal influenza in order to reduce its associated morbidity.

There are several limitations of our study. First, because of the heightened awareness associated with nH1N1, there is the possibility that the influence of pandemic influenza publicity may have lowered the threshold for admission of nH1N1 cases when compared with SI cases. In our review, all children admitted to the hospital had valid reasons for hospital admission and these reasons were distributed equally between patients with nH1N1 and SI. As other measures to determine if the threshold for hospitalization differed during the pandemic period as compared to the prior two influenza seasons, we assessed the proportion of hospitalized patients directly admitted to the PICU and the proportion of children with laboratory-confirmed influenza-like illness observed in the emergency room admitted to the hospital. There was no difference in these proportions further suggesting that these admissions were likely justified. The proportion of children evaluated in the emergency room and subsequently admitted to the hospital in our cohort is comparable to what has been previously reported in the literature for SI [10].

Second, no conclusions can be made regarding the differences in the virulence of the strains of influenza virus from this paper as we were unable to compare strain types. Children admitted with SI in the 2007-8 and 2008-9 seasons were not significantly different in terms of demographic features, underlying medical conditions, clinical course, and outcomes; however, we recognize that influenza types and subtypes, which vary from season to season, have differential effects on morbidity and mortality [23-25]. Our results demonstrate that SI 2007-9 was at least as significant a cause of pediatric morbidity as nH1N1.

## Conclusions

Pandemic nH1N1 received considerable attention with strong media messages urging people to be vaccinated and encouraging improved infection control efforts. As our study strengthens the growing body of evidence demonstrating that SI results in significant morbidity in the pediatric population, we believe that the emphasis on influenza vaccination should be continued on an annual basis and not solely reserved for influenza pandemics.

## List of Abbreviations

All abbreviations are defined in the text where first used.

## Competing interests

The authors declare that they have no competing interests.

## Authors' contributions

PDT performed data collection, analysis, and manuscript preparation. AT assisted with regression models. AMM and SEC critically reviewed the manuscript. AV was involved with the laboratory methods and ensured all nH1N1 strains were confirmed by PCR. AB provided a detailed database of all influenza positive patients including proportions of those children in the emergency department subsequently admitted to the hospital. TMP conceived the idea of the study and was involved in design and manuscript preparation. All authors read and approved the final draft.

## Pre-publication history

The pre-publication history for this paper can be accessed here:

http://www.biomedcentral.com/1471-2431/10/72/prepub

## References

[B1] Dawood FS Jain S Finelli L Shaw MW Lindstrom S Garten RJ Gubareva LV Xu X Bridges CB Uyeki TM Emergence of a novel swine-origin influenza A (H1N1) virus in humans N Engl J Med 2009 360 25 2605 2615 10.1056/NEJMoa0903810 19423869

[B2] Gojovic MZ Sander B Fisman D Krahn MD Bauch CT Modelling mitigation strategies for pandemic (H1N1) 2009 CMAJ 2009 181 10 673 80 19825923 10.1503/cmaj.091641 PMC2774362

[B3] Towers S Feng Z Pandemic H1N1 influenza: predicting the course of a pandemic and assessing the efficacy of the planned vaccination programme in the United States Euro Surveill 2009 14 4119358 19883540

[B4] Centers for Disease Control and Prevention Interim results: Influenza A (H1N1) 2009 monovalent vaccination coverage - United States, October-December 2009. MMWR Morb Mortal Wkly Rep 2010 59 2 44 48 20094027

[B5] Apisarnthanarak A Apisarnthanarak P Cheevakumjorn B Mundy LM Implementation of an infection control bundle in a school to reduce transmission of influenza-like illness during the novel influenza A 2009 H1N1 pandemic Infect Cont Hosp Epidemiol 2010 31 3 310 311 10.1086/651063 20109073

[B6] HHS Announces Nationwide Effort to Encourage H1N1 Vaccination During National Influenza Vaccination Week January 10 - 16, 2010 Accessed September 18^th ^2010.

[B7] Bhat N Wright JG Broder KR Murray EL Greenberg ME Glover MJ Likos AM Posey DL Klimov A Lindstrom SE Balish A Medina MJ Wallis TR Guarner J Paddock CD Shieh WJ Zaki SR Sejvar JJ Shay DK Harper SA Cox NJ Fukuda K Uyeki TM Influenza Associated Deaths among Children in the United States, 2003-2004 N Engl J Med 2005 353 24 2559 2667 10.1056/NEJMoa051721 16354892

[B8] Thompson WW Shay DK Weintraub E Brammer L Bridges CB Cox NJ Fukuda K Influenza-Associated Hospitalizations in the United States JAMA 2004 292 11 1333 1340 10.1001/jama.292.11.1333 15367555

[B9] Izurieta HS Thompson WW Kramarz P Shay DK Davis RL DeStefano F Influenza and the rates of hospitalization for respiratory disease among infants and young children N Engl J Med 2000 342 4 232 9 10.1056/NEJM200001273420402 10648764

[B10] Ampofo K Gesteland PH Bender J Mills M Daly J Samore M Byrington C Pavia AT Srivastava R Epidemiology, complications, and cost of hospitalization in children with laboratory-confirmed influenza infection Pediatrics 2006 118 6 2409 17 10.1542/peds.2006-1475 17142526

[B11] Langley JM Bradlley JS Defining pneumonia in critically ill infants and children Pediatr Crit Care Med 2005 6 3 Suppl S9 S13 10.1097/01.PCC.0000161932.73262.D7 15857566

[B12] CDC protocol of realtime RTPCR for influenza A H1N1 http://www.who.int/csr/resources/publications/swineflu/realtimeptpcr Updated April 30, 2009. Accessed September 1^st^, 2010

[B13] Coffin SE Zaoutis TE Rosenquist AB Heydon K Herrera G Bridges CB Watson B Localio R Hodinka RL Keren R Incidence, complications, and risk factors for prolonged stay in children hospitalized with community acquired influenza Pediatrics 2007 119 4 740 8 10.1542/peds.2006-2679 17403845

[B14] Neuzil KM Zhu Y Griffin MR Edwards KM Thompson JM Tollefson SJ Wright PF Burden of interpandemic influenza in children younger than 5 years: a 25-year prospective study J Infect Dis 2002 185 2 147 152 10.1086/338363 11807687

[B15] Neuzil KM Mellen BG Wright PF Mitchel EF Griffin MR The effect of influenza on hospitalizations, outpatient visits, and courses of antibiotics in children N Engl J Med 2000 342 225 31 10.1056/NEJM200001273420401 10648763

[B16] Simonsen L Clarke MJ Williamson GD Stroup DF Arden NH Schonberger LB The impact of influenza epidemics on mortality: introducing a severity index Am J Public Health 1997 87 12 1944 50 10.2105/AJPH.87.12.1944 9431281 PMC1381234

[B17] Glezen WP Payne AA Snyder DN Downs TD Mortality and influenza J Infect Dis 1982 146 3 313 21 7108280 10.1093/infdis/146.3.313

[B18] Matheson NJ Harnden AR Perera R Sheikh A Symmonds-Abrahams M Neuraminidase inhibitors for preventing and treating influenza in children Cochrane Database Syst Rev 2007 24 1 CD002744 10.1002/14651858.CD002744.pub2 17253479

[B19] Influenza vaccination coverage among children aged 6 months-18 years - eight immunization information system sentinel sites, United States, 2008-09 influenza season MMWR Morb Mortal Wkly Rep 2009 58 38 1059 1062 19798018

[B20] Fiore AE Shay DK Broder K Iskander JK Uyeki TM Mootrey G Bresee JS Cox NJ Prevention and control of seasonal influenza with vaccines: recommendations of the Advisory Committee on Immunization Practices (ACIP), 2009 MMWR Morb Mortal Wkly Rep 2009 58 RR08 1 52 19644442

[B21] Monto AS Kioumehr F The Tecumseh study of respiratory illness. IX. Occurence of influenza in the community, 1966--1971 Am J Epidemiol 1975 102 6 553 563 1202957 10.1093/oxfordjournals.aje.a112193

[B22] Glezen WP Decker M Perrotta DM Survey of underlying conditions of persons hospitalized with acute respiratory disease during influenza epidemics in Houston, 1978-1981 Am Rev Respir Dis 1987 136 3 550 555 3631727 10.1164/ajrccm/136.3.550

[B23] Glezen WP Couch RB Taber LH Paredes A Allison JE Frank AL Aldridge C Epidemiologic observations of influenza B virus infections in Houston, Texas, 1976-1977 Am J Epidemiol 1980 111 1 13 22 7352453 10.1093/oxfordjournals.aje.a112865

[B24] Frank AL Taber LH Glezen WP Geyer EA McIlwain S Paredes A Influenza B virus infections in the community and the family The epidemics of 1976-1977 and 1979-1980 in Houston, Texas. Am J Epidemiol 1983 118 3 313 25 10.1093/oxfordjournals.aje.a113638 6613976

[B25] Glezen WP Serious morbidity and mortality associated with influenza epidemics Epidemiol Rev 1982 4 25 44 6754408 10.1093/oxfordjournals.epirev.a036250

